# Effects of Auto-Titrating Mandibular Advancement Device on Autonomic Nervous System in Obstructive Sleep Apnea

**DOI:** 10.3390/jpm14121151

**Published:** 2024-12-13

**Authors:** Dae-Soon Son, Jae-In Kim, Dong-Kyu Kim

**Affiliations:** 1 Department of Data Science and Data Science Convergence Research Center, Hallym University, Chuncheon 24252, Republic of Korea; 2 Department of Physiology, Neurology, Hallym University College of Medicine, Chuncheon 24252, Republic of Korea; 3 Department of Otorhinolaryngology-Head and Neck Surgery, Chuncheon Sacred Heart Hospital, Hallym University College of Medicine, Chuncheon 24252, Republic of Korea; 4 Institute of New Frontier Research, Division of Big Data and Artificial Intelligence, Chuncheon Sacred Heart Hospital, Hallym University College of Medicine, Chuncheon 24252, Republic of Korea

**Keywords:** sleep, apnea, autonomic, mandibular advancement devices, orthodontic appliances

## Abstract

**Background/Objectives:** One prior study revealed that a newly developed auto-titrating mandibular advancement device (AMAD) could potentially enhance polysomnographic outcomes in individuals with obstructive sleep apnea (OSA). However, evidence regarding its impact on autonomic nervous system dysregulation in OSA remains limited. In this study, we aimed to compare the effects of conventional mandibular advancement devices (MADs) and AMDA on autonomic function. **Methods**: We retrospectively reviewed data from patients who visited a sleep center with complaints of snoring and sleep apnea (30 and 15 patients in the conventional MAD and AMAD groups, respectively). We assessed heart rate variability (HRV) frequency-domain metrics such as total power (TP), very low frequency (VLF), low frequency (LF), and high frequency (HF) using ultra-short-term and short-term modalities, assessing sympathetic and parasympathetic activity changes across treatment groups. **Results**: Conventional MAD treatment was associated with reductions in LF and LF/HF ratios, whereas AMAD treatment was linked to decreases in TP, VLF, LF, and LF/HF ratios. Notably, in patients with moderate OSA, LF values were significantly lower in the AMAD group than in the conventional MAD group. **Conclusions**: These findings suggest that both devices could reduce sympathetic over-activity in patients with OSA, with AMAD demonstrating greater efficacy, particularly in those with moderate OSA.

## 1. Introduction

Obstructive sleep apnea (OSA) is characterized by repeated upper airway obstruction, leading to intermittent hypoxia, fluctuations in intrathoracic pressure, and sleep disruption. The recurrent episodes of apnea and hypopnea during sleep trigger intermittent hypoxia, significant intrathoracic pressure fluctuations, and the activation of the sympathetic branch of the autonomic nervous system (ANS). The ANS, composed of sympathetic and parasympathetic branches, plays a critical role in regulating physiological states, particularly blood pressure. Consequently, dysregulated autonomic function, particularly increased sympathetic activity, is central to the development of hypertension [1,2,3,4]. OSA further affects blood pressure, as patients typically exhibit cyclical fluctuations in heart rate and blood pressure patterns. Such fluctuations are caused by a combination of chemoreflex-driven sympathetic activation and altered ventricular filling due to intrathoracic pressure shifts during OSA episodes. They are associated with heightened sympathetic activation and parasympathetic variability, contributing to the increased risk of hypertension and cardiovascular disease [5,6,7,8].

Managing OSA involves a multifaceted approach tailored to each patient. Continuous positive airway pressure (CPAP) remains the gold-standard treatment for moderate-to-severe OSA in adults [9,10]; however, its effectiveness is undermined by low adherence rates. To address this issue, a mandibular advancement device (MAD) was introduced as an alternative treatment modality and can be used as a first-line option for patients with simple snoring, mild-to-moderate OSA with low BMI, or increased upper airway resistance syndrome [11]. MAD therapy has become popular because of its superior convenience and patient compliance [11]. For instance, MADs offer specific advantages over CPAP, such as not requiring electricity and being more convenient to use in different sleep environments, making it a viable option for patients who do not improve with CPAP therapy or have poor adherence.

**Figure 1:**
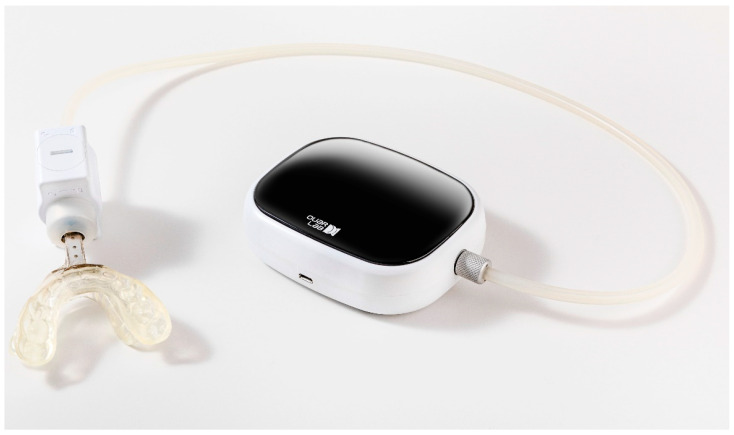
The design and structure of the auto-titrating mandibular advancement device.

Conventional MAD works by protruding the mandible during sleep to maintain upper airway patency. Recently, OUaR LaB, Inc. (Seoul, Republic of Korea) developed a novel type of MAD called the auto-titrating mandibular advancement device (AMAD) system, which features an innovative mechanism that automatically adjusts the degree of mandibular advancement based on the patient’s sleep position, increasing the degree when the patient is lying on their back and reducing it when they are on their side (Figure 1). This dynamic adjustment enhances its therapeutic efficacy for OSA and reduces the risk of side effects typically associated with static mandibular traction during sleep. A recent clinical trial indicated that AMAD may provide an effective alternative treatment for OSA, significantly improving various respiratory parameters, including the apnea–hypopnea index (AHI), lowest oxygen saturation, and arousal index [12]. Furthermore, no severe side effects such as hypersalivation, dental discomfort, mucosal dryness, or jaw pain were reported during the short-term application of AMAD. However, evidence to confirm improvements in ANS dysregulation with successful AMAD therapy for OSA remains insufficient. Heart rate variability (HRV) is known to reflect ANS balance and has implications for cardiovascular health [13]. Additionally, night-time HRV could serve as a valuable metric for assessing the effectiveness of MAD in treating OSA [14].

We proposed that conventional MAD and AMAD might have distinct effects on enhancing cardiac autonomic function in patients with OSA. Thus, this study aimed to evaluate and compare the impact of conventional MAD and AMAD therapies on autonomic nervous system function, measured through an HRV analysis.

## 2. Materials and Methods

### 2.1. Study Population

We retrospectively reviewed data from patients who visited a sleep center with complaints of snoring and sleep apnea. The inclusion criteria were as follows: (1) patients aged ≥18 years; (2) patients diagnosed with mild-to-moderate OSA (AHI between 5 and 30 events per hour); (3) patients treated with either a conventional MAD (SomnoDent; SomnoMed Ltd., Crows Nest, NSW, Australia) or AMAD (Oxleep™; OUaR LaB, Inc., Republic of Korea); and (4) patients who underwent follow-up polysomnography (PSG) 6 months after initiating treatment, with both baseline and post-MAD data available, and were identified as therapy responders. The exclusion criteria were (1) patients with a history of conditions known to affect HRV (e.g., cardiac arrhythmias, myocardial infarction, diabetic neuropathy, cardiac transplantation, myocardial dysfunction, tetraplegia); (2) patients with low-quality PSG raw data, such as artifacts affecting more than 20% of total sleep time; (3) patients with concurrent sleep disorders, such as insomnia or narcolepsy; (4) chronic users of sedatives or hypnotics; and (5) patients classified as non-responders to treatment.

Anthropometric data were collected for all participants, and their levels of daytime sleepiness and subjective sleep quality were assessed using standardized questionnaires: the Epworth Sleepiness Scale (ESS) for measuring daytime drowsiness and the Pittsburgh Sleep Quality Index (PSQI) for evaluating overall sleep quality. We also collected and compared baseline characteristics between the two groups, offering insights into the differences in patient characteristics and sleep metrics before treatment initiation. These baseline comparisons are crucial for understanding the initial conditions and validating subsequent evaluations of treatment effects. The study adhered to ethical standards outlined by the institutional research committee and complied with the 1964 Declaration of Helsinki and its subsequent amendments, ensuring the protection of participants’ rights and safety. Ethical approval was obtained from the Institutional Review Board (IRB) of Hallym University Chuncheon Sacred Hospital (IRB No. 2024-01-012, date: 12 January 2024). All data used in this study were fully included in the analysis. Additional data may be available for further review upon request, pending institutional approval and compliance with relevant data protection regulations.

### 2.2. Sleep Study

A standard overnight PSG was conducted at the Chuncheon Sacred Hospital sleep laboratory using a computerized system (Nox-A1; Nox Medical, Reykjavik, Iceland). Each participant underwent an 8 h overnight PSG monitoring session while in bed. Participants were instructed to avoid alcohol, caffeine, naps, and prolonged or intense physical activity on the day of the study. During PSG monitoring, we gathered physiological data such as electroencephalogram, electromyogram, electrooculogram, electrocardiogram, and oxygen saturation levels; body movements; and nasal airflow. We scored sleep stages and respiratory events according to the American Academy of Sleep Medicine guidelines [15]. The sleep parameters assessed included the total sleep time (TST), sleep efficiency (TST divided by time in bed, expressed as a percentage), oxygen desaturation index (ODI), and AHI. ODI refers to the number of oxygen desaturation episodes per hour of sleep, with desaturation defined as >3% reduction in blood oxygen saturation from the baseline. Apnea was detected with an oronasal thermal sensor when the signal decreased by ≥90% from the baseline for at least 10 s. Hypopnea was recorded when nasal pressure signal excursions decreased by ≥30% from the baseline for at least 10 s, accompanied by a ≥3% drop in oxygen saturation or arousal. AHI represents the combined total events of apneas and hypopneas per hour of sleep. OSA severity was classified based on AHI: no OSA (AHI < 5 per hour), mild OSA (AHI between 5 and 15 per hour), moderate OSA (AHI between 15 and 30 per hour), and severe OSA (AHI ≥ 30 per hour). Successful MAD treatment was defined as >50% reduction in AHI, with AHI dropping below 10 events per hour while using the MAD compared to the baseline value. Additionally, a treatment response was defined as >50% reduction in AHI, with AHI falling below 20 events per hour when using MAD compared to baseline values [16]. Participants who did not achieve these thresholds were classified into the non-response group.

### 2.3. Assessment of Autonomic Function

HRV refers to fluctuations in the time interval between consecutive heartbeats, typically measured through RR intervals from an ECG signal. HRV represents the balance between the parasympathetic and sympathetic nervous systems. A nocturnal HRV analysis is widely used as a noninvasive assessment of autonomic function across various fields [17]. Evidence also shows that HRV can evaluate changes in ANS activity and may be a valuable indicator of OSA severity [18,19,20]. In this study, we extracted electrocardiography data from PSG recordings to ensure accuracy and quality through visual inspection. We removed ectopic beats and artifacts and included only normal-to-normal beats in the HRV analysis. Time-domain HRV parameters reflect the variability in beat-to-beat intervals, affecting sympathetic and parasympathetic nervous system activity. However, these parameters are generally not used to differentiate the balance between sympathetic and parasympathetic functions. Therefore, we focused on frequency-domain HRV parameters. We applied a spectral power analysis by shifting predetermined segments (ultra-short term of 2 min and short term of 5 min) forward by 2 s throughout the entire 10 min HRV dataset collected from each patient. The frequency-domain parameters were total power (TP), representing power at frequencies ≤ 0.4 Hz; very low frequency (VLF), at frequencies ≤ 0.04 Hz; low frequency (LF), between 0.04 and 0.15 Hz; and high frequency (HF), between 0.15 and 0.4 Hz. The LF/HF ratio was calculated by dividing the LF by the HF. Normalized LF power (LFnu) is expressed as [LF/(LF + HF) × 100], whereas normalized HF power (HFnu) is expressed as [HF/(LF + HF) × 100]. In the time-domain HRV analysis, short-term segments of HRV were generally measured over 5 min, with the magnitude of frequency components derived from the spectral analysis being valuable for evaluating ANS activity. However, the HRV analysis using short-term segments has inherent limitations in clinical practice, as its spectral estimates cannot capture rapid ANS fluctuations. Consequently, ultra-short-term segmentation (lasting less than 5 min) has recently been introduced across various medical disciplines, including sleep medicine [19].

### 2.4. Statistical Analysis

Most variables collected in this study were continuous variables reported as the mean ± standard deviation, with the exception of sex, which was a categorical variable represented as a ratio. To compare the baseline characteristics of each treatment group (conventional MAD and AMAD groups), we used independent *t* tests or chi-square tests. To assess the differences between baseline parameters and those after MAD treatment, a paired *t* test was used. Welch’s *t* test was performed to compare HRV parameters between the conventional MAD and AMAD groups. All statistical analyses were performed using R software for Windows (version 4.0.0; R Foundation for Statistical Computing, Vienna, Austria). Based on the G power program, in the present study, the sample size was calculated based on an effect size of 0.7, a parent distribution assumed to follow a Laplace distribution, and an alpha error of 0.05. All *p* values were two-sided, with statistical significance at *p* < 0.05 in two-tailed tests.

## 3. Results

### 3.1. Patient Characteristics

Following the inclusion and exclusion criteria, 30 participants were allocated to the conventional MAD group and 15 to the AMAD group. Baseline demographic characteristics, clinical profiles, and overnight polysomnographic findings for all participants are shown in Table 1. No statistically significant differences were found between the conventional MAD and AMAD groups in terms of baseline characteristics such as sex, age, body mass index, ESS, and PSQI. Similarly, no significant differences were observed between the two groups in baseline PSG parameters, including TST, sleep latency, sleep efficiency, AHI, and ODI.

### 3.2. Changes in Nocturnal Heart Rate Variability According to the Different Types of Mandibular Advancement Devices

Among patients receiving conventional MAD treatment, the LF value demonstrated significant reductions in ultra-short-term (15,052.29 ± 1057.50) and short-term (15,061.61 ± 1031.05) HRV analyses. Additionally, the short-term HRV analysis revealed a marked increase in VLF values (24,087.84 ± 7524.17) in these patients (Table 2). Similarly, in patients undergoing AMAD treatment, significant reductions were observed in TP, VLF, LF, and LF/HF ratios in both ultra-short-term (43,356.15 ± 7398.55, 19,137.63 ± 7398.55, 13,645.90 ± 797.45, and 2.38 ± 0.50) and short-term (41,268.15 ± 6843.13, 22,638.98 ± 4620.36, 14,090.57 ± 709.80, and 2.59 ± 0.59) HRV analyses. However, no significant changes were observed in HF values in patients treated with AMAD across both the ultra-short-term and short-term HRV assessments (Table 3).

### 3.3. The Comparison of the Changes in Nocturnal Heart Rate Variability According to the Severity of Obstructive Sleep Apnea

In patients diagnosed with mild OSA, no statistically significant differences were identified in any of the HRV parameters when comparing the conventional MAD group to the AMAD group, as demonstrated in Table 4. These findings suggest that both devices may exert similar effects on ANS function in this subgroup of patients with milder forms of OSA. However, a different trend was observed in patients with moderate OSA. In this group, LF levels were notably and significantly lower in the AMAD group than in the conventional MAD group. Specifically, the LF levels were 13,354.77 ± 637.35 in the ultra-short term and 13,870.29 ± 757.24 in the short term, as shown in Table 5. This finding highlights the potential differences in the physiological impact between the two devices, particularly in patients with more pronounced disease severity.

## 4. Discussion

Although the exact underlying mechanisms linking OSA and cardiovascular complications remain poorly understood, it is well established that patients with OSA often exhibit increased sympathetic nervous system activity and alterations in ANS function. These changes in the ANS may contribute to the cardiovascular risks associated with OSA. While various treatments have been explored for their impact on ANS function, it is important to note that the specific effects of AMAD therapy on ANS dysregulation have not yet been thoroughly investigated. Therefore, in this study, we sought to explore the differential effects of conventional MAD and AMAD therapies on ANS function by utilizing HRV measurements as an indicator of ANS activity. Additionally, we used ultra-short-term HRV features, which have been increasingly applied across various medical fields to address the limitations of short-term HRV measures [19,21,22,23], to assess ANS function in patients with OSA along with short-term HRV features. Our findings showed that MAD therapy significantly altered cardiac autonomic modulation, as reflected by nocturnal HRV, with more pronounced changes in patients with moderate OSA treated with AMAD compared to those treated with conventional MAD.

OSA is a common sleep-breathing disorder associated with disturbances in the ANS function. PSG parameters and sleep questionnaires are widely recognized as classical and representative methods for evaluating the efficacy of treatments for OSA. These tools are valuable in assessing various aspects of sleep quality and treatment effectiveness. However, despite their usefulness, they are limited in their ability to provide insights into ANS function. While PSG and sleep questionnaires measure sleep architecture and subjective experiences, they do not capture the complex physiological changes associated with ANS regulation, which is critical in understanding the broader impact of OSA treatments on cardiovascular and autonomic health. Therefore, additional evaluation methods, such as HRV measurements, are necessary to assess ANS function in this context.

HRV serves as a crucial noninvasive marker for assessing autonomic regulation. HRV is usually described by frequency-domain measurements, such as HF and LF. The HF components of HRV indicate parasympathetic activity, whereas the LF components reflect sympathetic activity [18]. Several studies have confirmed that patients with OSA exhibit increased sympathetic activity and reduced parasympathetic function. Typically, sympathetic activity decreases, and parasympathetic activity increases during sleep. However, in patients with OSA, ANS alterations, particularly sympathetic over-activity, occur both acutely during apnea events and chronically during the daytime. These disruptions contribute to the cardiovascular consequences of sleep-disordered breathing. In healthy controls, the ANS functions in a well-coordinated manner during sleep; however, in patients with OSA, sympathetic nerve impairment results in an incomplete suppression of sympathetic activity, which is typically inhibited during sleep. Respiratory events may also trigger abnormal sympathetic activation, contributing to an abnormal elevation in the LF values. Consequently, the LF frequency-domain index may be a more sensitive marker for detecting sympathetic nerve impairments during sleep in patients with OSA.

A recent meta-analysis revealed that patients with OSA exhibited significantly elevated LF power and an increased LF/HF ratio during nocturnal periods, indicating reduced parasympathetic activity [24]. Additionally, the LF/HF ratio demonstrates significant robustness in patients with OSA, suggesting its potential as a valuable tool in the future diagnosis of the condition [24]. Moreover, the relationship between OSA and sympathetic dysregulation appears to be dose-dependent, with evidence suggesting that sympathetic over-activity may be reversible upon the initiation of OSA treatment [2,3]. Consistent with these findings, our study revealed a prominent improvement in LF values in patients with moderate OSA treated with AMAD compared to those treated with conventional MAD. Previous studies also show that age, sex, and BMI can significantly influence HRV in healthy individuals. However, in this study, no significant differences in these variables were observed between the conventional MAD and AMAD groups [25,26,27].

This study has some limitations. First, we acknowledge the small sample size as a limitation of our study. Because this was a pilot study evaluating a new medical device, ethical considerations limited the inclusion of a larger cohort. Future studies with larger and more diverse populations are needed to confirm our findings. Second, unfortunately, due to the retrospective nature of the data and our focus on comparing two active treatments, we did not include a control group in this study. Furthermore, our IRB does not ethically permit the use of a placebo treatment with no proven medical efficacy as a control group for patients who visit for treatment purposes. We believe that this is a fundamental limitation of medical device research in general. Therefore, to overcome this limitation, we included a group of patients using a conventional MAD instead of a control group in this study. Third, the presence of comorbidities may have influenced the inflammatory state in patients with OSA, potentially acting as a confounding factor in the HRV analysis [28,29,30,31]. Fourth, only time-domain HRV parameters were evaluated to assess ANS function, as frequency-domain HRV parameters require longer RR interval segments for an accurate analysis. Fifth, although we matched the major independent variables, including sex, age, and BMI, between the two groups, our study did not include HRV data from control subjects, limiting comparative insights. Sixth, we relied solely on linear methods for HRV feature extraction without incorporating non-linear approaches. Future studies should compare linear and non-linear methodologies. Lastly, further investigation is needed to assess the long-term effects of MAD treatment in patients with OSA.

## 5. Conclusions

Considering that the effects of AMAD on ANS dysregulation have not been studied, in this study, we examined the differential effects of conventional MAD and AMAD therapies on ANS function using HRV measurements. By examining these differences, we wanted to provide valuable insights into how these therapeutic approaches may influence ANS regulation and, consequently, cardiovascular health in patients with OSA. Notably, we found that MAD use alters cardiac autonomic modulation, particularly by reducing sympathetic activity. Moreover, AMAD had a more pronounced effect in lowering sympathetic parameters compared to conventional MAD in patients with moderate OSA. This effect was not observed in patients with mild OSA, suggesting a potential association between MAD treatment and the reduction in cardiac autonomic dysregulation, which may help reduce cardiovascular risk. Additionally, the use of AMAD could provide enhanced patient outcomes compared to conventional MAD, paving the way for more personalized treatment approaches.

## Figures and Tables

**Table 1:** Baseline characteristics and laboratory overnight polysomnographic parameters.

	Conventional MAD (n = 30)	AMAD (n = 15)	*p* Value
Sex (male, %)	26 (86.7)	12 (80.0)	0.88
Age (years)	53.9 ± 7.65	55.07 ± 10.67	0.71
BMI (kg/m^2^)	24.77 ± 1.85	24.13 ± 2.80	0.44
ESS	6.57 ± 2.25	6.27 ± 1.39	0.06
PSQI	7.10 ± 2.92	7.13 ± 2.0	0.96
**Baseline PSG**			
Total sleep time (min)	361.17 ± 58.01	343.13 ± 60.69	0.21
Sleep latency (min)	44.43 ± 8.15	42.73 ± 12.30	0.63
Sleep efficiency (%)	80.40 ± 6.68	74.93 ± 6.65	0.06
AHI	14.77 ± 9.13	14.13 ± 6.23	0.79
ODI	8.57 ± 5.68	7.93 ± 5.12	0.71

Data are expressed as mean ± standard deviation. AHI, apnea–hypopnea index; AMAD, auto-titrating mandibular advancement device; BMI, body mass index; ESS, Epworth Sleepiness Scale; ODI, oxygen desaturation index; PSG, polysomnography; PSQI, Pittsburgh Sleep Quality Index.

**Table 2:** Efficacy of conventional mandibular advancement device (MAD) treatment on shift in autonomic dysregulation in patients with obstructive sleep apnea.

n = 30	Baseline	Conventional MAD Treatment	Effect Size	*p* Value
**Ultra-short-term**				
TP (ms^2^)	71,417.84 ± 91,198.39	44,059.44 ± 8304.53	0.4219 (0.1006, 0.9444)	0.11
VLF (ms^2^)	31,665.46 ± 8280.15	24,407.97 ± 20,520.27	0.4638 (−0.0599, 0.9876)	0.07
LF (ms^2^)	17,018.89 ± 386.35	15,052.29 ± 1057.50	2.4703 (1.784, 3.1565)	<0.01
HF (ms^2^)	5836.69 ± 1332.13	5942.05 ± 1220.21	−0.0825 (−0.5995, 0.4346)	0.75
LF/HF ratio	3.07 ± 0.72	2.63 ± 0.53	0.6984 (0.1160, 1.2307)	<0.01
LFnu	74.73 ± 4.14	71.92 ± 3.81	0.7010 (0.1685, 1.2335)	<0.01
HFnu	25.27 ± 4.14	28.08 ± 3.81	−0.7010 (−1.2335, −0.1685)	<0.01
**Short-term**				
TP (ms^2^)	67,694.21 ± 65,636.94	42,875.24 ± 8304.53	0.5305 (0.0047, 1.0564)	0.05
VLF (ms^2^)	31,052.73 ± 11,032.64	24,087.84 ± 7524.17	0.7376 (0.2035, 1.2717)	<0.01
LF (ms^2^)	16,984.13 ± 564.34	15,061.61 ± 1031.05	2.1332 (1.6455, 2.9808)	<0.01
HF (ms^2^)	5740.93 ± 1516.62	5565.60 ± 1256.20	0.1259 (−0.3914, 0.6433)	0.63
LF/HF ratio	3.17 ± 0.81	2.83 ± 0.59	0.4738 (−0.0502, 0.9979)	0.07
LFnu	75.09 ± 4.74	73.29 ± 3.94	0.4152 (−0.1072, 0.9375)	0.11
HFnu	24.91 ± 4.74	26.71 ± 3.94	−0.4152 (−0.9375, 0.1072)	0.11

Data are expressed as mean ± standard deviation. Total power (TP), representing power at frequencies ≤ 0.4 Hz; very low frequency (VLF), power at frequencies ≤ 0.04 Hz; low frequency (LF), power between 0.04 and 0.15 Hz; high frequency (HF), power between 0.15 and 0.4 Hz; LF/HF ratio, calculated as LF divided by HF; LFnu, normalized LF power expressed as [LF/(LF + HF) × 100]; and HFnu, normalized HF power expressed as [HF/(LF + HF) × 100].

**Table 3:** Efficacy of auto-titrating mandibular advancement device (AMAD) treatment on shift in autonomic dysregulation in patients with obstructive sleep apnea.

n = 15	Baseline	AMAD Treatment	Effect Size	*p* Value
**Ultra-short-term**				
TP (ms^2^)	69,783.72 ± 5488.07	43,356.15 ± 7398.55	4.2437 (2.8950, 5.5923)	<0.01
VLF (ms^2^)	31,447.63 ± 5147.10	19,137.63 ± 7398.55	2.4203 (1.4359, 3.4047)	<0.01
LF (ms^2^)	16,954.95 ± 345.16	13,645.90 ± 797.45	5.3855 (3.7769, 6.9942)	<0.01
HF (ms^2^)	5681.07 ± 1287.25	5981.98 ± 1324.00	−0.2304 (−0.9809, 0.5200)	0.06
LF/HF ratio	3.12 ± 0.67	2.38 ± 0.50	1.2613 (0.4423, 2.0803)	<0.01
LFnu	75.16 ± 4.00	69.80 ± 4.42	1.2691 (0.4493, 2.0890)	<0.01
HFnu	24.84 ± 4.00	30.20 ± 4.42	−1.2691 (−2.0890, −0.4493)	<0.01
**Short-term**				
TP (ms^2^)	68,490.45 ± 5488.07	41,268.15 ± 6843.13	4.3896 (3.0086, 57,705)	<0.01
VLF (ms^2^)	31,624.46 ± 5629.82	22,638.98 ± 4620.36	1.7448 (0.8660, 2.6236)	<0.01
LF (ms^2^)	16,945.74 ± 534.53	14,090.57 ± 709.80	4.5442 (3.1288, 5.9597)	<0.01
HF (ms^2^)	5485.34 ± 1447.02	5733.05 ± 1394.67	−0.1743 (−0.9237, 0.5751)	0.64
LF/HF ratio	3.29 ± 0.83	2.59 ± 0.59	0.9743 (0.1832, 1.7654)	<0.01
LFnu	75.85 ± 4.66	71.40 ± 4.71	0.9481 (0.1593, 1.7370)	<0.01
HFnu	24.15 ± 4.66	28.60 ± 4.71	−0.9481 (−1.7370, −0.1593)	<0.01

Data are expressed as mean ± standard deviation. Total power (TP), representing power at frequencies ≤ 0.4 Hz; very low frequency (VLF), power at frequencies ≤ 0.04 Hz; low frequency (LF), power between 0.04 and 0.15 Hz; high frequency (HF), power between 0.15 and 0.4 Hz; LF/HF ratio, calculated as LF divided by HF; LFnu, normalized LF power expressed as [LF/(LF + HF) × 100]; and HFnu, normalized HF power expressed as [HF/(LF + HF) × 100].

**Table 4:** Comparison of nocturnal heart rate variability between conventional mandibular advancement device (MAD) and auto-titrating mandibular advancement device (AMAD) groups in patients with mild obstructive sleep apnea (OSA).

Mild OSA	Conventional MAD	AMAD	Test Statistics	Effect Size	*p* Value
**Ultra-short-term**					
TP (ms^2^)	44,111.93 ± 8624.79	43,348.00 ± 9114.64	0.20	−0.0872 (−0.9474, 0.7730)	0.84
VLF (ms^2^)	20,441.30 ± 6506.23	18,898.35 ± 5102.89	0.67	−0.2505 (−1.1131, 0.6122)	0.51
LF (ms^2^)	14,663.74 ± 872.95	13,900.63 ± 874.49	2.09	−0.8738 (−1.7665, 0.0190)	0.06
HF (ms^2^)	5641.62 ± 1137.80	5929.12 ± 1108.35	−0.62	0.2544 (−0.6083, 1.1172)	0.55
LF/HF ratio	2.70 ± 0.56	2.41 ± 0.44	1.4311	−0.5410 (−1.4136, 0.3317)	0.17
LFnu	72.41 ± 3.90	70.25 ± 3.91	1.32	−0.5535 (−1.4267, 0.3197)	0.21
HFnu	27.59 ± 3.90	29.75 ± 3.91	−1.32	0.5535 (−0.3197, 1.4267)	0.21
**Short-term**					
TP (ms^2^)	43,220.77 ± 7432.44	40,994.05 ± 8183.73	0.67	−0.2914 (−1.1550, 0.5722)	0.52
VLF (ms^2^)	24,347.97 ± 7145.90	21,784.67 ± 4026.35	1.20	−0.3970 (−1.2638, 0.4697)	0.24
LF (ms^2^)	14,725.13 ± 865.56	14,283.31 ± 652.04	1.47	−0.5430 (−1.4158, 0.3297)	0.16
HF (ms^2^)	5221.29 ± 1144.04	5630.11 ± 996.72	−0.94	0.3695 (−0.4963, 1.2354)	0.36
LF/HF ratio	2.94 ± 0.60	2.60 ± 0.43	1.66	−0.6009 (−1.4765, 0.2746)	0.11
LFnu	74.04 ± 3.99	71.87 ± 3.51	1.42	−0.5606 (−1.4341, 0.3130)	0.18
HFnu	25.96 ± 3.99	28.13 ± 3.51	−1.42	0.5606 (−0.3130, 1.4341)	0.18

Data are expressed as mean ± standard deviation. Total power (TP), representing power at frequencies ≤ 0.4 Hz; very low frequency (VLF), power at frequencies ≤ 0.04 Hz; low frequency (LF), power between 0.04 and 0.15 Hz; high frequency (HF), power between 0.15 and 0.4 Hz; LF/HF ratio, calculated as LF divided by HF; LFnu, normalized LF power expressed as [LF/(LF + HF) × 100]; and HFnu, normalized HF power expressed as [HF/(LF + HF) × 100]. Continuous variables were assessed using Welch’s *t* test, along with corresponding *t* statistic and degrees of freedom (df).

**Table 5:** Comparison of nocturnal heart rate variability between conventional mandibular advancement device (MAD) and auto-titrating mandibular advancement device (AMAD) groups in patients with moderate obstructive sleep apnea (OSA).

Moderate OSA	Conventional MAD	AMAD	Test Statistics	Effect Size	*p* Value
**Ultra-short-term**					
TP (ms^2^)	43,954.48 ± 11,698.87	43,365.47 ± 5549.79	0.14	−0.0872 (−0.9474, 0.7730)	0.89
VLF (ms^2^)	32,341.30 ± 34,096.28	19,411.10 ± 5325.58	1.18	−0.2505 (−1.1131, 0.6122)	0.27
LF (ms^2^)	15,829.38 ± 994.09	13,354.77 ± 637.35	6.25	−0.8738 (−1.7665, 0.0190)	<0.01
HF (ms^2^)	6542.91 ± 1209.49	6042.38 ± 1627.61	0.69	0.2544 (−0.6083, 1.1172)	0.51
LF/HF ratio	2.49 ± 0.47	2.34 ± 0.60	0.56	−0.5410 (−1.4136, 0.3317)	0.59
LFnu	70.95 ± 3.60	69.29 ± 5.23	0.73	−0.5535 (−1.4267, 0.3197)	0.49
HFnu	29.05 ± 3.60	30.71 ± 5.23	−0.73	0.5535 (−0.3197, 1.4267)	0.49
**Short-term**					
TP (ms^2^)	42,184.17 ± 10,237.44	41,581.40 ± 555.48	0.16	−0.2914 (−1.1550, 0.5722)	0.88
VLF (ms^2^)	23,567.59 ± 8612.04	23,615.34 ± 5367.75	−0.01	−0.3970 (−1.2638, 0.4697)	0.99
LF (ms^2^)	15,734.57 ± 1043.54	13,870.29 ± 757.24	4.27	−0.5430 (−1.4158, 0.3297)	<0.01
HF (ms^2^)	6254.22 ± 1237.50	5850.70 ± 1830.10	0.51	0.3695 (−0.4963, 1.2354)	0.62
LF/HF ratio	2.60 ± 0.50	2.57 ± 0.77	0.09	−0.6009 (−1.4765, 0.2746)	0.93
LFnu	71.79 ± 3.56	70.88 ± 6.07	0.36	−0.5606 (−1.4341, 0.3130)	0.73
HFnu	28.21 ± 3.56	29.12 ± 6.07	−0.36	0.5606 (−0.3130, 1.4341)	0.73

Data are expressed as mean ± standard deviation. Total power (TP), representing power at frequencies ≤ 0.4 Hz; very low frequency (VLF), power at frequencies ≤ 0.04 Hz; low frequency (LF), power between 0.04 and 0.15 Hz; high frequency (HF), power between 0.15 and 0.4 Hz; LF/HF ratio, calculated as LF divided by HF; LFnu, normalized LF power expressed as [LF/(LF + HF) × 100]; and HFnu, normalized HF power expressed as [HF/(LF + HF) × 100]. Continuous variables were assessed using Welch’s *t* test, along with corresponding t statistic and degrees of freedom (df).

## Data Availability

All pertinent data supporting the conclusions of this study are provided in the analysis. Additional data may be requested for further review with access contingent on institutional approval and data protection guidelines.
